# Combining flagellin and human β-defensin-3 to combat bacterial infections

**DOI:** 10.3389/fmicb.2014.00673

**Published:** 2014-12-09

**Authors:** Ofra Sabag, Haya Lorberboum-Galski

**Affiliations:** Department of Biochemistry and Molecular Biology, Institute for Medical Research Israel-Canada, Hebrew University–Hadassah Medical SchoolJerusalem, Israel

**Keywords:** antibacterial peptide β-defensin-3 (BD3), flagellin (F), fusion protein (FBD3), bacterial infection, antibacterial activity, TLR5 receptor, human CD4+ T cells

## Abstract

The discovery and therapeutic use of antibiotics made a major contribution to the reduction of human morbidity and mortality. However, the growing resistance to antibiotics has become a matter of huge concern. In this study we aimed to develop an innovative approach to treat bacterial infections utilizing two components: the human antibacterial peptide β-defensin-3 (BD3) and the bacterial protein flagellin (F). This combination was designed to provide an efficient weapon against bacterial infections with the peptide killing the bacteria directly, while the flagellin protein triggers the immune system and acts against bacteria escaping from the peptide’s action. We designed, expressed and purified the fusion protein flagellin BD3 (FBD3) and its two components, the F protein and the native BD3 peptide. FBD3 fusion protein and native BD3 peptide had antibacterial activity *in vitro* against various bacterial strains. FBD3 and F proteins could also recognize their receptor expressed on target cells and stimulated secretion of IL-8. In addition, F and FBD3 proteins had a partial protective effect in mice infected by pathogenic *Escherichia coli* bacteria that cause a lethal disease. Moreover, we were able to show partial protection of mice infected with *E. coli* using a flagellin sequence from *Salmonella*. We also explored flagellin’s basic mechanisms of action, focusing on its effects on CD4+ T cells from healthy donors. We found that F stimulation caused an increase in the mRNA levels of the Th1 response cytokines IL12A and IFNγ. In addition, F stimulation affected its own receptor.

## INTRODUCTION

The effectiveness of antibiotics, used since Fleming’s discovery in 1927 to fight bacterial infections, has decreased in recent years due to the emergence of “superbugs” – bacterial strains resistant to all known antibiotic treatments. This is considered to be one of the world’s most critical health problems, and is believed to be a consequence of the frequent overuse of antibiotic agents. Moreover, according to FDA reports, the development and research of new antibiotic drugs has declined in the recent years (Taubes, 2008). For these reasons, the design of new strategies for the development of anti-bacterial agents has become of major interest.

One of the most promising fields in medicine today is biological drugs. Among these drugs, fusion proteins are considered to be a powerful tool for targeted treatment of diseases such as cancers, autoimmune diseases or metabolic diseases (Lorberboum-Galski, 2011). Here, for the first time, we used this powerful tool as a new approach to treating bacterial infections, utilizing two components: the human antibacterial peptide β-defensin-3 (BD3) that belongs to the innate immune system and the bacterial protein flagellin that induces the immune system. This combination is hypothesized to provide an efficient weapon to fight and combat bacterial infections; while the peptide will kill the bacteria directly, the flagellin, recognized by its receptor-TLR5, will trigger the immune system and locally act against the residual bacteria that succeed in escaping the peptide’s action.

To test this concept we used two strategies; the first was to design a fusion protein composed of both molecules – one moiety being the flagellin protein and the other an antibacterial peptide – fused at the complementary DNA (cDNA) level. The second strategy was to use the two components individually.

The innate immune system is the body’s first line of defense against various pathogens. The recognition of invader microbes by the host is done through a series of receptors among them the Toll-like receptor (TLR) family is the best characterized (Kawai and Akira, 2007). These receptors can recognize limited and highly conserved sets of molecular structures produced by microorganisms that are termed pathogen associated molecular patterns (PAMPs; Mogensen, 2009). The recognition of a particular PAMP by a specific TLR leads to a cascade that eventually causes activation of the transcription factor NFκB (Kawai and Akira, 2010). TLR5 can recognize a site on the highly conserved bacterial protein flagellin (Andersen-Nissen et al., 2007). Flagellin has four globular domains: D0, D1, D2, and D3, with domain D1 containing the highly conserved regions involved in flagellin recognition and signaling (Yonekura et al., 2003). The monomer form of flagellin induces TLR5 signaling (Smith et al., 2003).

One important component belonging to the innate immune system is the antibacterial peptides. These peptides are highly heterogeneous in sequence, length and structure but most of them are small and positively charged with the ability to kill a broad-spectrum of bacteria, fungi, yeasts, and viruses (Jenssen et al., 2006). One of the most promising antibacterial peptides is the BD3; a 45-residue cationic peptide with an asymmetrical distribution of charged amino acids, mostly clustered at the carboxyl-terminal region. BD3 shows a broad spectrum of antibacterial activity against Gram-negative and Gram-positive bacteria, including resistant strains (Shelburne et al., 2005).

To test our hypothesis, we chose the gene *fliC* coding for *Salmonella typhimurium* flagellin, and as an antibacterial peptide we used the gene coding for BD3. By fusing these two moieties we obtained the fusion protein flagellin BD3 (FBD3). We also cloned and produced the two components separately; the native BD3 peptide (nBD3) and the flagellin protein (F).

The new antibacterial fusion protein and the separate recombinant proteins/peptides were tested for their ability to target and kill bacteria in both *in vitro* and *in vivo* models. We also explored basic mechanisms in which flagellin is involved, focusing on its effects on the subpopulation of CD4+ T cells from healthy donors.

## MATERIALS AND METHODS

### CELL CULTURES AND BACTERIAL STRAIN

#### Cell cultures

Colo-205, human colon cancer carcinoma cells (ATCC, USA, catalog no. CCL-222) and Bjab-human, Burkitt’s lymphoma cells B blast, (kindly provided by Hanna Ben-Bassat, Hadassah Medical Center, Jerusalem) cell lines were grown in Roswell Park Memorial Institute (RPMI) medium (Biological Industries, Beit-Haemek, Israel) supplemented with 10% fetal bovine serum (HyClone, Logan, UT, USA), 100 μg/ml penicillin/streptomycin and 2 mM L-glutamine (Biological Industries).

#### Bacterial strains

*Escherichia coli* DH5 alpha, *Salmonella typhimurium* (ATCC 14028), *E. coli* (ATCC 25922) and *Staphylococcus aureus* (ATCC 6538) were obtained from ATCC (Manassas, VA, USA).

### CONSTRUCTION OF PLASMIDS EXPRESSING FLAGELLIN AND β-DEFENSIN-3 PROTEINS

#### Flagellin

To obtain the *Salmonella* flagellin sequence total DNA from *Salmonella* lysate was isolated and the flagellin sequence (aa 1–495) flanked by *Nde* I/*Bam*H I restriction sites was obtained by polymerase chain reaction (PCR) using the following primers:

5′-CGCCATATGGCACAAGTCATTAAT-3′ (sense)

5′-CGGGATCCTTACGCAGTAAAGAGAG-3′ (antisense)

The PCR product cut with *Nde* I/*Bam*H I was cloned into a pET28 expression vector, cut with the same restriction enzymes, so obtaining the pOS01 plasmid.

#### Flagellin β-defensin-3

To construct the fusion protein the same 5′ primer for flagellin was used (see Flagellin) with the following antisense primer to obtain the flagellin sequence:

5′-CGGGATCCACGCAGTAAAGAGAGGAC-3′ (antisense)

The PCR product cut with *Nde* I/*Bam*H I was cloned into a *Nde* I/*Bam*H I cut pET28 expression vector. The next step was to obtain the *BD3* sequence flanked with *Bam*H I/*Hin*d III. The cDNA for human *BD3* was obtained by RT-PCR, using RNA isolated from fresh phytohaemagglutinin (PHA) activated human lymphocytes. Total RNA was isolated and reverse transcribed into cDNA, using a reverse transcription kit (Promega, Madison, WI, USA). The *BD3* (aa 1–45), flanked by *Bam*H I/*Hin*d III restriction sites was obtained by PCR using the following primers:

5′-CCCCATATGCAGAAATATTATTGCAGA-3′ (sense)

5′-CCCAAGCTTTTATTTCTTTCTTCGGCA-3′ (antisense)

The PCR product cut with *Bam*H I/*Hin*d III was cloned into a cut pET28 expression vector downstream to flagellin sequence (see above), so obtaining the pOS03 plasmid.

#### Native β-defensin-3

To construct the native peptide the same cDNA and the same 5′ primer for *BD3* were used (see above) with the following antisense primer to obtain the *BD3* sequence: 5′-CCATGGGCATCATAAACACATTA-3′ (antisense). The PCR product cut with *Nde* I/*Bam*H I was cloned into a pET28 expression vector, cut with the same restriction enzymes so obtaining the pOS04 plasmid. All the clones were confirmed by sequencing analysis.

### PROTEIN EXPRESSION, PURIFICATION OF THE RECOMBINANT PROTEINS AND ITS CHARACTERIZATIONS

#### Proteins expressions and purifications

*Escherichia coli* BL21-CodonPlus (λDE3) competent cells transformed with the plasmids encoding the recombinant proteins/peptide F, FBD3, and nBD3 were incubated at 37°C in a saline lactose broth (SLB) medium containing kanamycin (50 μg/ml), tetracycline (12.5 μg/ml), and chloramphenicol (34 μg/ml). At an OD_600_ of 0.8, protein expression was induced by adding isopropyl-beta-D-thiogalactopyranoside (IPTG, 1 mM final concentration). After 2 h of incubation at 37°C, the cells were harvested by centrifugation (500 g for 15 min at 4°C). For the purification procedure of F and FBD3, bacteria pellets were sonicated in binding buffer [PBS – pH 7.4, 1 mM phenylmethylsulfonyl fluoride (PMSF) and 40 mM imidazole (Sigma-Aldrich, St. Louis, MO, USA)]. The suspensions were clarified by centrifugation (35,000 g for 30 min at 4°C), and the supernatants containing the recombinant proteins were purified, using pre-equilibrated HiTrap chelating HP columns (Amersham-Pharmacia Biotech, Uppsala, Sweden). Columns were washed by stepwise addition of increasing imidazole concentrations. Finally, the target proteins were eluted with elution buffer (PBS – pH 7.4 + 500 mM imidazole). FBD3 was dialyzed against PBS, aliqoted and kept at -20°C.

For the F protein, an additional purification step was performed to remove endotoxins. The purified F protein was dialyzed against MES [(2-(*N*-morpholino)ethanesulfonic acid)] buffer (20 mM Tris-HCl, pH 5.5) and loaded onto SP-Sepharose FF (1.5Å∼5 cm) equilibrated with MES buffer. The recombinant protein was eluted with MES buffer supplemented with 0.5 M NaCl, dialyzed against PBS aliqoted and kept at -20°C.

For the purification of nBD3, bacteria pellets were sonicated in binding buffer (MES – pH 5.5, 1 mM PMSF and 0.1 M NaCl). The suspensions were clarified by centrifugation (35,000 g for 30 min at 4°C), and the supernatants containing the recombinant peptide were purified using SP-Sepharose FF (1.5Å∼5 cm) pre-equilibrated with MES buffer. The recombinant peptide was eluted with MES buffer supplemented with 0.8 M NaCl. The purified peptide was dialyzed against various buffers and found to be highly active in 20 mM Tris pH -8.

#### Characterization of the fusion proteins

***Determination of protein concentration***. Protein concentration was measured according to the Bradford method, using the Bradford reagent and a standard curve of bovine serum albumin (BSA). Protein concentration was determined at a wavelength of 595 nm.

***Separation of proteins by electrophoresis***. Samples from the various protein fractions (5–20 μg protein/lane) were loaded on 12% (w/v) SDS/PAGE gels for the proteins or tricine gel for the peptides (Judd, 1994).

***Western blot analysis***. Proteins and peptides (5–20 μg protein/lane) were resolved respectively on 12% SDS-PAGE and tricine gels and transferred onto an Immobilon-P transfer membrane (Millipore, Bradford, PA, USA). Western blot analysis was performed using either anti-His (Amersham-Pharmacia Biotech), or anti-β defensin 3 (Santa-Cruz Biotechnology) antibodies at 1:20,000 or 1:200 dilution respectively, to identify the relevant proteins/peptides. Primary antibody binding was detected by blotting with a suitable secondary antibody conjugated to horseradish peroxidase (HRP; 1:10,000). Band visualization was performed using an enhanced chemiluminescence kit (EZ-ECL, Biological Industries, Beit-Haemek, Israel) according to manufacturer’s instructions.

### ACTIVITY ASSAYS

#### Antibacterial activity assay

Glycerol stocks from various pathogenic bacteria were spread on agar plates and incubated overnight at 37°C. One colony from the plate was removed to fresh Luria-Broth (LB) medium for an overnight growing starter. The overnight bacterial starter was diluted 1/100 with fresh LB medium and different amounts of the tested proteins/peptide were added to the diluted bacteria and then grown at 37°C for 3 h. The growing bacteria were serially diluted and 5 μl from each dilution was plated on LB agar plates. The LB agar plates were incubated at 37°C overnight and on the next day the number of colony forming units (CFU) was determined.

#### Flagellin activity assay

Bjab or Colo-205 cells were seeded on 6-well plates. After 24 h the fusion protein FBD3 (0.2–20 ng), the recombinant protein F (0.1–12 ng), or GnRH-Cherry (20 ng) as a control protein were added to the cells. After 4 h incubation the cell-free supernatants were analyzed according to the manufacturer’s instructions for human IL-8 content, using Human IL-8 ELIZA KIT II (BD Biosciences San Diego, CA, USA). The wells were spectrophotometrically analyzed at 450 nm using a VersaMax microplate reader (Molecular Devices). The concentration of each unknown sample was determined according to a standard curve of IL-8 concentrations and analyzed using the four-parameter algorithm on SoftMax Pro software (Molecular Devices Corporation, CA, USA).

### FLOW CYTOMETRY

To verify the amount of TLR5 expressed on the cell surface of the various cell cultures (Colo-205, Bjab and CD4+ T cells), the cells (0.5–1 × 10^6^ cells in total) were washed twice with PBS, centrifuged at 300 g for 5 min, resuspended in 50 μl of the fluorescence-activated cell sorting (FACS) buffer (3% FCS, 0.02% Azide in PBS), and incubated for 30 min at 4°C with rabbit anti-TLR5 Ab (1:200; Alexis Plymouth Meeting, PA, USA). The cells were washed twice with FACS buffer, resuspended in 50 μl of the same buffer, and the human cell lines were incubated with FITC-conjugated rabbit anti-goat secondary Ab (Jackson ImmunoResearch Laboratories, Bar Harbor, ME, USA) for 20 min at 4°C. The cells were washed twice with FACS buffer, resuspended in 0.5 ml PBS, and analyzed by FACScan (Becton Dickinson Immunocytometry Systems, San Jose, CA, USA) using the CellQuest program. For the CD4+ T cells additional staining was performed with anti-human CD4-PE-conjugated (1:50; Serotec, Oxford, UK). The cells were washed twice with FACS buffer, resuspended in 0.5 ml PBS, and analyzed by FACScan.

### ISOLATION OF PERIPHERAL BLOOD MONONUCLEAR CELLS (PBMCs) FROM HEALTHY DONORS

The human peripheral blood mononuclear cells (PBMCs) from healthy donors were isolated on a Ficoll–Isopaque gradient (*d* 1.077 Sigma-Aldrich, St. Louis, MO, USA). The isolated PBMCs were then counted and checked for viability using Trypan blue. To activate the lymphocytes, all mononuclear cells were grown in RPMI 1640 supplemented with 10% (v/v) fetal calf serum, 2 mM L-glutamine, 100-units/ml penicillin, 100 μg/ml streptomycin, 10 μM β-mercaptoethanol and with phytohemagglutinin (PHA 20 μg/ml) for 3 days. The activated cells were washed and resuspended in the presence of 10 units/ml of recombinant IL-2 (PeproTech EC Ltd., London, UK) to maintain cell viability. Naïve lymphocytes were grown in RPMI 1640 supplemented with 10% (v/v) fetal calf serum, 2 mM L-glutamine, 100 units/ml penicillin, 100 μg/ml streptomycin and 10 μM β-mercaptoethanol.

### FLAGELLIN EXPERIMENTS

#### Experiments on isolated CD4+ T cells

CD4+ T cells were isolated from human fresh mononuclear cells by CD4+ starting kit (Miltenyi Biotec Auburn, CA, USA) and were 95–98% pure according to FACS analysis. Following purification, CD4+ T cells (10^6^ cells) were plated out and incubated in the presence or absence of the purified F protein (2 ng/ml), for 18 or 24 h, once or three times. In some experiments the fresh mononuclear cells were activated by PHA and were grown with IL2 (see above). Following activation, CD4+ T cells were isolated with the same kit and incubated in the presence or absence of the F protein as described above.

#### Experiments on PBMCs cells

Peripheral blood mononuclear cells (10^7^ cells) were seeded on plates and incubated in the presence or absence of F protein (2 ng/ml or 6 ng/ml) for 2, 6, 12, 24, or 36 h. Following incubation the CD4+ T cells were separated using the CD4+ starting kit (see above) and were 95–98% pure according to FACS analysis.

### RNA ISOLATION AND REAL-TIME PCR

Total RNA was extracted from the various treated cells using the High Pure RNA Isolation Kit (Roche, Germany). The RNA was reverse transcribed using a Verso cDNA kit (Thermo Scientific, Epson, Surrey, UK) using random primers. Real-time quantitative PCR was performed on an ABI Prism 7500 HT sequence detection system using SYBR® Green methods (Applied Biosystems, Foster City, CA, USA). All these steps were carried out according to the manufacturer’s instructions. Specific gene expression was normalized to that of the G6PD gene. Relative expression of IL4, IL12A, IFNγ, and TLR5 mRNA levels in treated cells as compared to control cells was calculated using the 2^-Ct^ analysis (Applied Biosystems). All samples were run in triplicate (**Table 1**).

**Table 1:** Primers used for testing expression levels of specific genes by real time PCR.

Gene	Location	Product size	Sequence
G6PD	Exon 3/4	129 bp	Forward 5′-CACCATCTGGTGGCTGTTC-3′
	Exon 4		Backward 5′-TCACTCTGTTTGCGGATGTC-3′
IFNγ	Exon 2/3	100 bp	Forward 5′- TTGGAAAGAGGAGAGTGACAGAAA-3′
	Exon 3		Backward 5′-CTTTTGGATGCTCTGGTCATCTTTA-3′
IL12A	Exon 2	151 bp	Backward 5′-GAATGTTCCCATGCCTTCAC-3′
	Exon 2/3		Forward 5′-TCTAGAGTTTGTCTGGCCTTCTG-3′
IL4	Exon 3	278 bp	Backward 5′-GCAGTTCCACAGGCACAAG-3′
	Exon 4		Forward 5′-CTCTGGTTGGCTTCCTTCAC-3′
IL5	Exon 1/2	192 bp	Backward 5′-GCCAATGAGACTCTGAGGATTC-3′
	Exon 3		Forward 5′-CCCTTGCACAGTTTGACTCTC-3′
TLR5	Exon 3/4	261 bp	Forward 5′-TGCTAGGACAACGAGGATCA-3′
	Exon 4		Backward 5′-CAGGAAGGAATTCCAAACACA-3′

### *IN VIVO* MODEL

All the experiments were performed using 7–8 week old female Balb/C mice in the specific pathogen-free unit of the Hadassah Medical School (Ein Kerem, Jerusalem). For testing the non-specific toxicity of F and FBD3, 5 or 10 μg of F or 20 μg of FBD3 were administered i.p. to 7–8 week old female Balb/C mice. Mice were checked daily and weighed every 1 or 2 days.

To determine the CFU of pathogenic *E. coli* (ATCC 25922) that was needed to cause mice death, a series of dilutions were made and different concentrations of bacteria were injected i.p. into mice in 200 μl of sterile PBS. To test the effect of F and FBD3 on infected mice, 5 (eight mice) or 10 μg of F (10 mice) or 20 μg (10 mice) of FBD3 were injected into 7–8 week old female Balb/C mice. Four hours later 5 × 10^8^ CFU/ml of *E. coli* bacteria (ATCC 25922) in 200 μl of sterile PBS were injected i.p. into these mice. Mice were checked for mortality every day.

### ETHIC STATEMENT

Fresh buffy coat human blood samples from healthy donors were obtained according to the guidelines approved by the Hadassah Hospital Ethics Committee (Jerusalem, Israel). All subjects provided written informed consent.

All procedures involving mice were approved by the Hebrew University of Jerusalem Institutional Animal Care and Use Committee, approval no. MD-07-10429-5. This study was conducted in accordance with the NIH Guide for the Care and Use of Laboratory Animals (NIH approval number OPRR-A01-5011). The mice were housed in cages with autoclaved bedding and equipped with filter caps under containment protocols with up to five animals per cage. A 12-h light/dark cycle was maintained, and mice had access to water and rodent laboratory chow *ad libitum*.

The ethics committee approved this whole study, including work on human samples as well as work on mice.

## RESULTS

### CONSTRUCTION AND EXPRESSION OF RECOMBINANT PROTEINS

**FIGURE 1:**
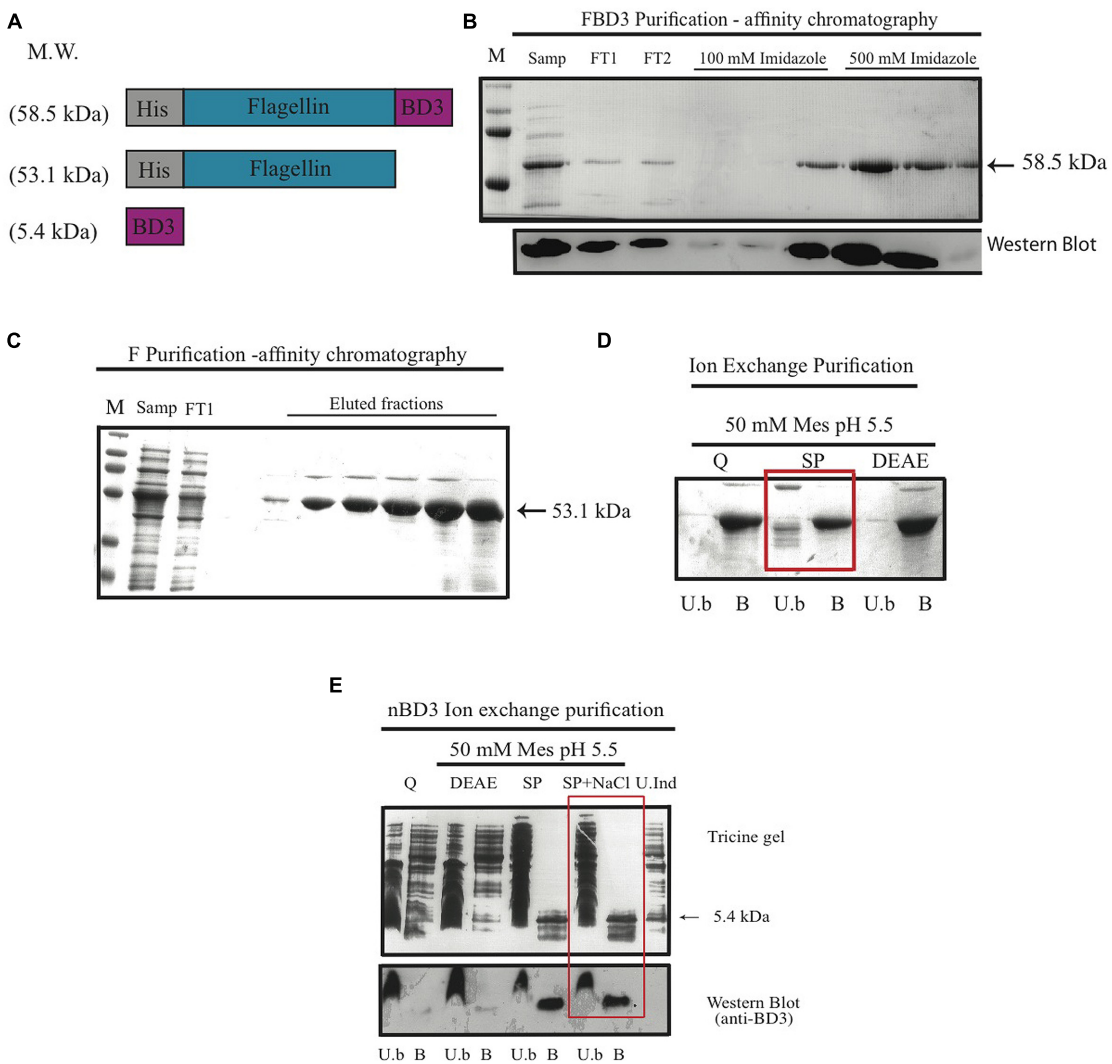
**The FBD3 fusion protein and the separate components; schematic presentation and purification. (A)** Schematic representation of flagellin β-defensin-3 (FBD3) fusion protein, flagellin (F) and the native BD3 (nBD3) lacking the His-Tag. **(B)** SDS-PAGE (upper image) and Western blot using anti-His antibodies (lower image) to analyze the purification steps of FBD3 using affinity chromatography as described in Methods. **(C)** SDS-PAGE of purification steps of F using affinity chromatography as described in Methods and using ion exchange chromatography **(D)** as described in Methods. The chosen resin for F purification is marked by a square. **(E)** Tricine gel and Western blot using anti-BD3 antibodies analysis of nBD3 purification steps using ion exchange chromatography as described in Methods. M, marker; W.C, whole cell extract; Sol, soluble fraction; InSol, insoluble fraction; Samp, sample; FT1/2, flow through1/2; U.b, unbound fraction; B, bound fraction; DEAE, diethylaminoethyl weak anion; SP, sulphopropyl strong cation; Q, quaternary amine strong anion, U.Ind, Un-induced fraction.

In order to test our hypothesis, we constructed the recombinant fusion protein FBD3 that contains the bacterial protein flagellin and the anti-bacterial peptide BD3. We used the full coding sequence of the gene *flic* from *Salmonella typhimurium* (aa 1–495) and the sequence of the human BD3, containing 45 amino acids of the mature peptide. The nBD3 was cloned 5′ to the flagellin sequence. We also constructed each moiety separately: flagellin (F) and nBD3. Apart from nBD3, the recombinant proteins were constructed with a Histidine (HisX6) tag at their N′ terminus. **Figure 1A** represents the schematic structures of the recombinant proteins/peptide and their expected molecular weights. All clones were expressed in several *E. coli* bacterial strains and under different conditions to obtain high expression levels. Upon expression, bacterial cells were disrupted and cellular sub-fractions were prepared, separating the soluble and non-soluble fractions. SDS-PAGE and Western blot analysis using anti-His or anti-beta defensin 3 antibodies demonstrated that the recombinant proteins were found mostly in the soluble fraction (results not shown).

Next we purified the various proteins using affinity chromatography (for FBD3 and F) and ion exchange (for nBD3 and F; **Figures 1B,C**). To remove endotoxin from the purified F protein, an additional purification step was performed using various ion exchange resins and buffers. As can be seen in **Figure 1D**, the SP resin gave the best results; while most of the undesired proteins did not bind to the resin, the F protein did bind to it (marked by a square).

To purify the nBD3 peptide, which lacked the His-Tag, we used conventional purification methods. Western blot analysis using anti-His (**Figure 1B**, lower image) or anti BD3 (**Figure 1E**; lower image) confirmed the identity of the FBD3 fusion protein and nBD3 peptide, respectively.

### FBD3, nBD3, AND F *IN VITRO* ACTIVITY

#### Antibacterial activity of nBD3 and FBD3

**FIGURE 2:**
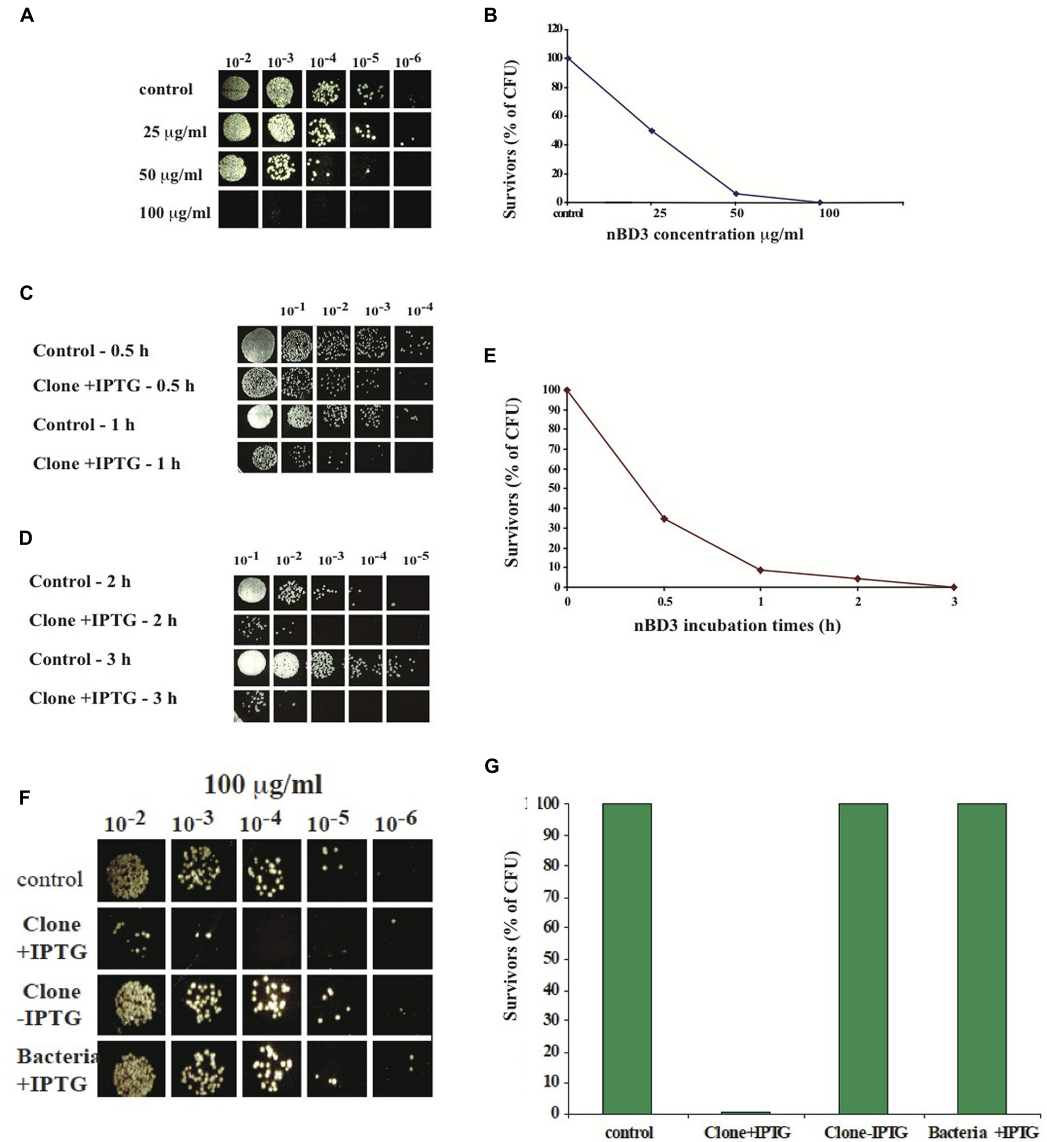
***In vitro* anti-bacterial activity of nBD3 peptide. (A,B)** *In vitro* anti-bacterial activity of nBD3 peptide against *Escherichia coli* (DH5α) bacteria – dose dependency. Antibacterial activity of the nBD3 peptide with increasing concentrations of the peptide. **(B)** The results in graphic presentation. One representative experiment out of four performed is shown. Variations were ±5%. **(C–E)** Kinetics of nBD3 peptide (100 μg/ml) anti-bacterial activity against DH5α *E. coli* bacteria. **(C,D)** *In vitro* antibacterial activity of the nBD3 peptide after 0.5, 1, 2, and 3 h against *E. coli* bacteria. **(E)** Graphic presentation of the results in **(C,D).** One representative experiment out of three performed is shown. Variations were ± 5%. **(F,G)** The nBD3 peptide has specific antibacterial activity against *E. coli* bacteria. **(F)** Effect of nBD3 and controls on bacterial growth. **(G)** Graphic presentation of results presented in **(F)**. Control: 20 mM Tris pH 8. Clone +IPTG: native BD3 peptide expressed in Codon+ BL21 *E. coli.* Clone –IPTG: Codon+ BL21 *E. coli* transformed with the plasmid for the BD3 peptide but without induction with IPTG. Bacteria +IPTG: Codon+ BL21 *E. coli* with induction of IPTG but without clone. One representative experiment out of four performed is shown. Variations were ±5%.

To analyze the antibacterial activity of the nBD3 peptide and the FBD3 fusion protein, we developed an *in vitro* assay that tests their effect on the CFU of different strains of growing bacteria. Cell survival was calculated by comparing the number of CFU of bacterial cells treated with the nBD3 peptide or the FBD3 fusion protein to those of control bacterial cells. We found that nBD3 was highly active and very efficiently killed *E. coli* bacteria (DH5α) in a dose-dependent manner. At the highest concentration (100 μg/ml) nBD3 decreased the survival rate to <0.2% (**Figures 2A,B**).

Next we tested the kinetics of the peptide action. We found that the antibacterial activity of nBD3 is time-dependent and after 3 h incubation, most of the bacteria did not survive (**Figures 2C–E**).

In order to confirm that the measured anti-bacterial activity is a result of the peptide’s action and not caused by non-specific activity of the purified peptide’s preparation, we used two controls: the first was a soluble fraction of *E. coli* BL21 Codon+ that was grown without the plasmid of the nBD3 clone (marked as bacteria +IPTG). This fraction was expressed and purified under the same conditions as were used for the nBD3. The second control was a soluble fraction of *E. coli* BL21 Codon+ that was grown with the plasmid of the nBD3 clone but without the inducer, IPTG (marked as Clone -IPTG). This sample was also expressed and purified under the same conditions as were used for the native peptide. As seen in **Figures 2F,G**, at 100 μg/ml of the different tested samples, only nBD3 (marked as clone +IPTG) had antibacterial activity and caused a decrease of 93% in the CFU, while the two control samples (Bacteria –IPTG and Clone –IPTG) showed no anti-bacterial killing activity.

We also tested the antibacterial activity of nBD3 on other strains of bacteria. We chose the following bacterial strains: *E. coli* (ATCC 25922); a pathogenic recommended and widely used gram-negative bacterium for antibiotic susceptibility assays. *Staphylococcus aureus* (ATCC 6538), which are pathogens of humans and other mammals. *Salmonella typhimurium* (ATCC 14028), a gram-negative bacterium causing systemic infections and typhoid fever in humans.

**FIGURE 3:**
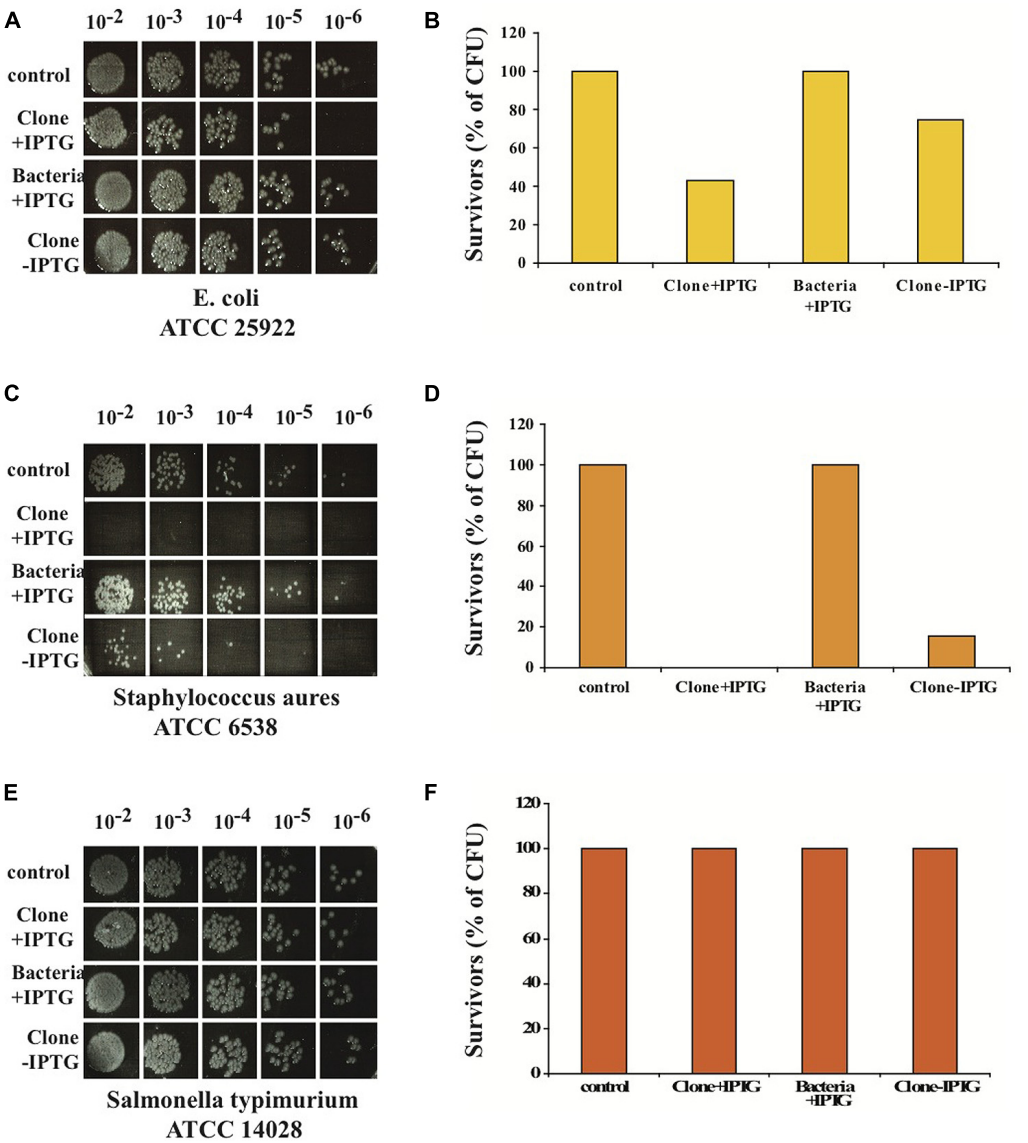
***In vitro* anti-bacterial activity of nBD3 peptide and control samples against different bacterial strains.** *In vitro* anti-bacterial activity of the nBD3 peptide (100 μg/ml) against: *E. coli* ATCC 25922 **(A)**, *Staphylococcus aureus* ATCC 6538 **(C)** and *Salmonella typhimurium* ATCC 14028 **(E)**. **(B,D,F)** Graphic presentations of results presented in **(A,C,E)**, respectively. One representative experiment out of four performed is shown.

We found that the nBD3 could kill pathogenic *E. coli* (ATCC 25922) and *Staphylococcus aureus* (ATCC 6538) but not *Salmonella typhimurium* (ATCC 14028; **Figures 3A–F**). The control samples (Bacteria –IPTG and Clone –IPTG) showed no killing activity against the pathogenic *E. coli* and *Staphylococcus aureus* bacterial strains (**Figures 3A–F**), confirming its specific anti-bacterial activity.

**FIGURE 4:**
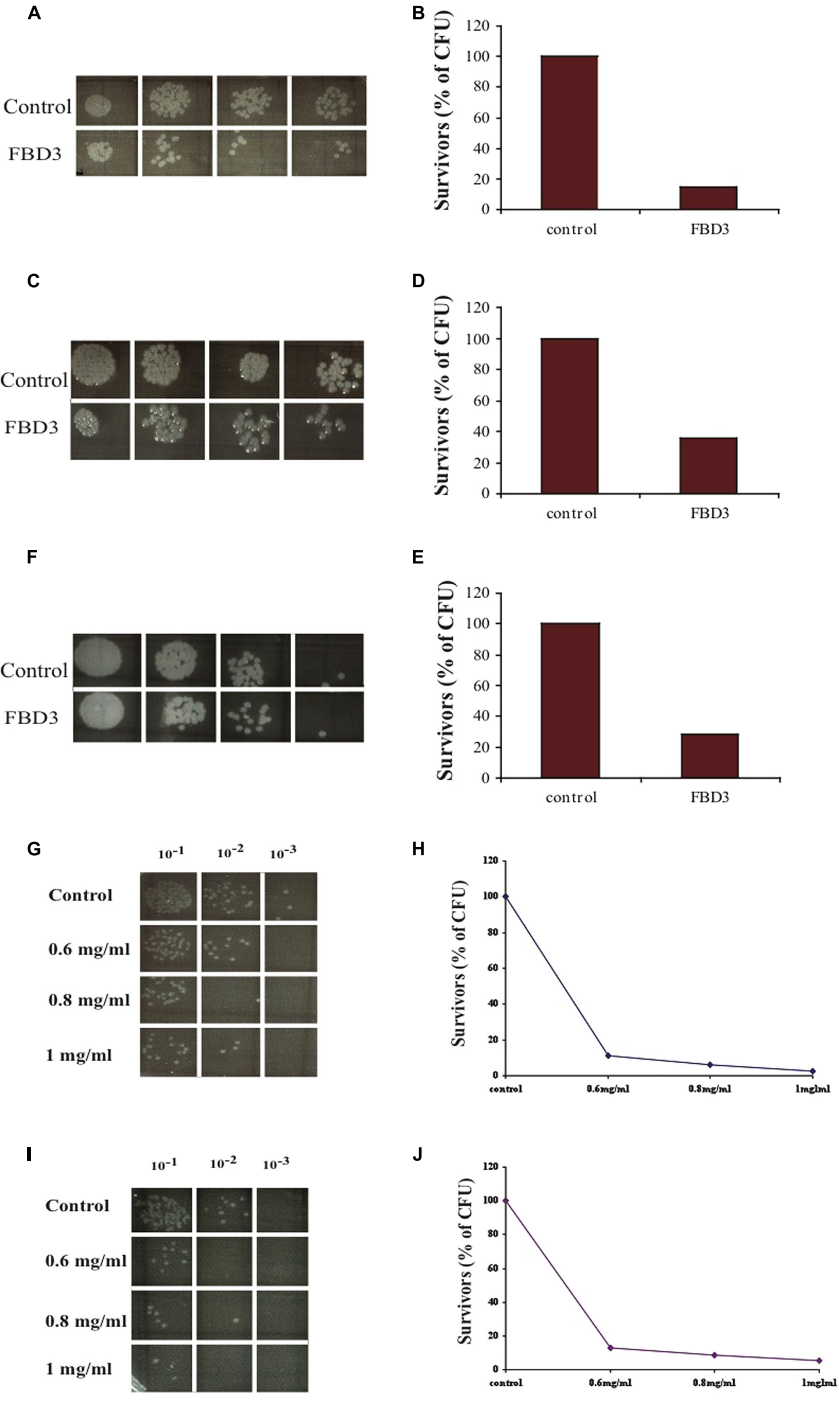
***In vitro* anti-bacterial activity of the FBD3 fusion protein. (A,C,E)** Antibacterial activity of three different expressions of purified FBD3 (0.2 mg/ml) against pathogenic *E. coli* (ATCC 25922). **(B,D,F)** represent the results of **(A,C,E)** respectively, in graphs. **(G–J)** Antibacterial activity at increasing concentrations of FBD3 in PBS with 10 mM DTT **(G)** or 50 mM DTT **(I). (H,J)** represent the results of **(G,J)** respectively, in graphs. One representative experiment out of four performed, is shown. Variations were ±5%.

In order to examine whether the FBD3 fusion protein possesses the antibacterial activity of the BD3 moiety, we used the *in vitro* antibacterial activity assay. It should be pointed out that the peptide moiety is 10-fold smaller than the flagellin protein, a fact that could be a limiting factor for its antibacterial activity. As seen in **Figures 4A–F**, the antibacterial moiety of FBD3 has activity against pathogenic *E. coli* and the survival rate of the bacteria was 20–40% when we added 0.2 mg/ml of FBD3 fusion protein (comprising about 20 μg/ml of the BD3 peptide component). However, although it had antibacterial activity *in vitro*, we encountered difficulties in obtaining biologically active and reproducible FBD3-fusion protein preparations. As already mentioned, defensin peptides have three intermolecular disulfide bonds. Based on site-directed mutagenesis studies, these bonds are not essential for the antibacterial activity of the peptide but are important for peptide stability and its chemotactic activity (Hoover et al., 2003; Wu et al., 2003). In an attempt to resolve these problems, we dialyzed the FBD3 against PBS in the presence of 10 mM or 50 mM DTT. As seen in **Figures 4G–J**, the fusion proteins that were dialyzed in the presence of DTT exhibited antibacterial activity and killed bacteria in a dose-dependent manner. At the highest concentration (1 mg/ml; comprising about 100 μg/ml of the BD3 peptide component), the FBD3 fusion protein decreased the bacterial survival rate to 5%.

#### The FBD3 fusion protein and the F protein exhibit flagellin biological activity

**FIGURE 5:**
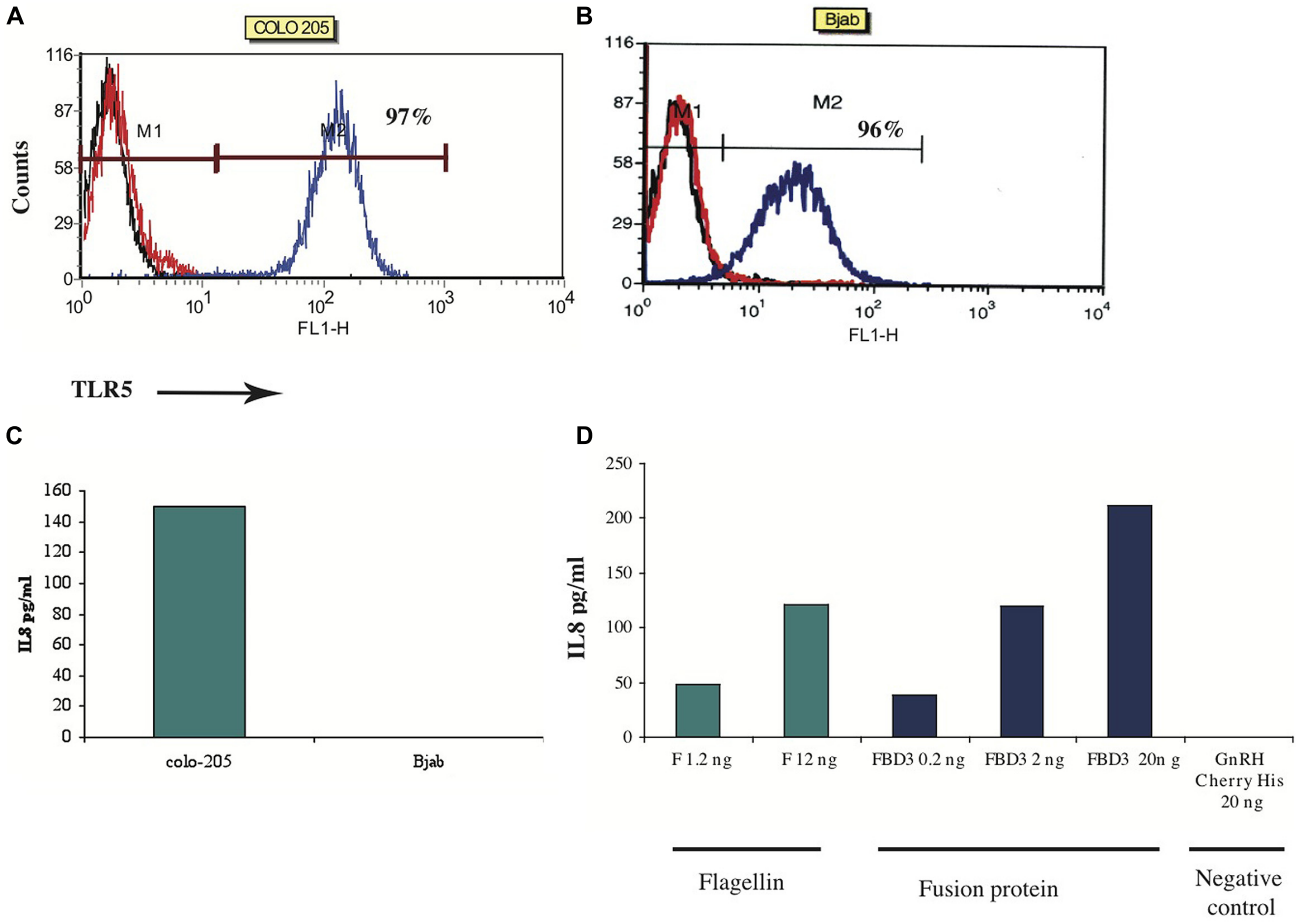
***In vitro* activity of F and FBD3 proteins-secretion of IL-8. (A,B)** FACS analysis of Toll-Like-Receptor-5 on Colo-205 and Bjab cell lines. **(C,D)** *In vitro* activity of the F protein on Colo-205 and Bjab cells. Colo-205 or Bjab cells (10^5^ cells/ml) were incubated with 20 ng (protein) of F for 4 h. The medium separated from the various samples was tested and the amount of the IL-8 was measured as described in Methods. One representative experiment out of two performed is shown **(C)**. Variations were ±10%. Colo-205 cells (10^5^ cells/ml) were incubated with different concentrations of F (1.2 and 12 ng protein), FBD3 (2 or 20 ng fusion protein; comprising about 1.8 and 18 ng, respectively, of the F protein) and GnRH-Cherry for 4 h (20 ng protein). The medium from the different samples was analyzed and the amount of the IL-8 was measured by an Elisa assay as described in the “Methods.” One representative experiment out of four performed is shown **(D)**.

Next we tested the flagellin moiety. In order to examine the biological activity of the flagellin, we designed an *in vitro* assay that measures one of flagellin’s activities – secretion of a chemokine upon binding to its TLR5 receptor. Based on the literature, colon cancer cells highly express TLR5 and can secrete the chemokine IL-8 upon stimulation with flagellin, while B cells express the receptor but cannot secrete this chemokine (Schmausser et al., 2005). To test the sensitivity of this assay, the expression of TLR5 was examined in a number of cell lines by FACS analysis. We found that colo-205 and Bjab cell lines do indeed highly express TLR5 (97%; mean: 127 and 96%; mean: 123 respectively; **Figures 5A,B**). To measure the levels of IL-8 secreted upon F stimulation, we used an ELISA kit for human IL-8. As seen in **Figure 5C**, colo-205 cells that were incubated for 4 h with 20 ng of the purified F protein secreted the chemokine IL-8, while Bjab cells did not secrete any detectable levels of IL-8.

Next we tested the activity of the flagellin moiety in the fusion protein FBD3. As seen in **Figure 5D**, FBD3 could trigger colo-205 cells to secrete IL-8 and its activity was dose-dependent, similar to the activity of the F protein (**Figure 5D**). A control protein, GnRH-Cherry, lacking the flagellin protein, expressed and purified under the same conditions as FBD3 and F, could not trigger these cells to secrete IL-8 even at high doses of the protein (**Figure 5D**). Moreover, addition of both FBD3 fusion protein and the F protein to cells negative for the expression of TLR5 receptor, could also not trigger these cells to secrete IL-8 (results not shown). Thus, the activity of the flagellin moiety of FBD3 is specific.

### FLAGELLIN PARTIALLY PROTECT MICE FROM BACTERIAL INFECTION

**FIGURE 6:**
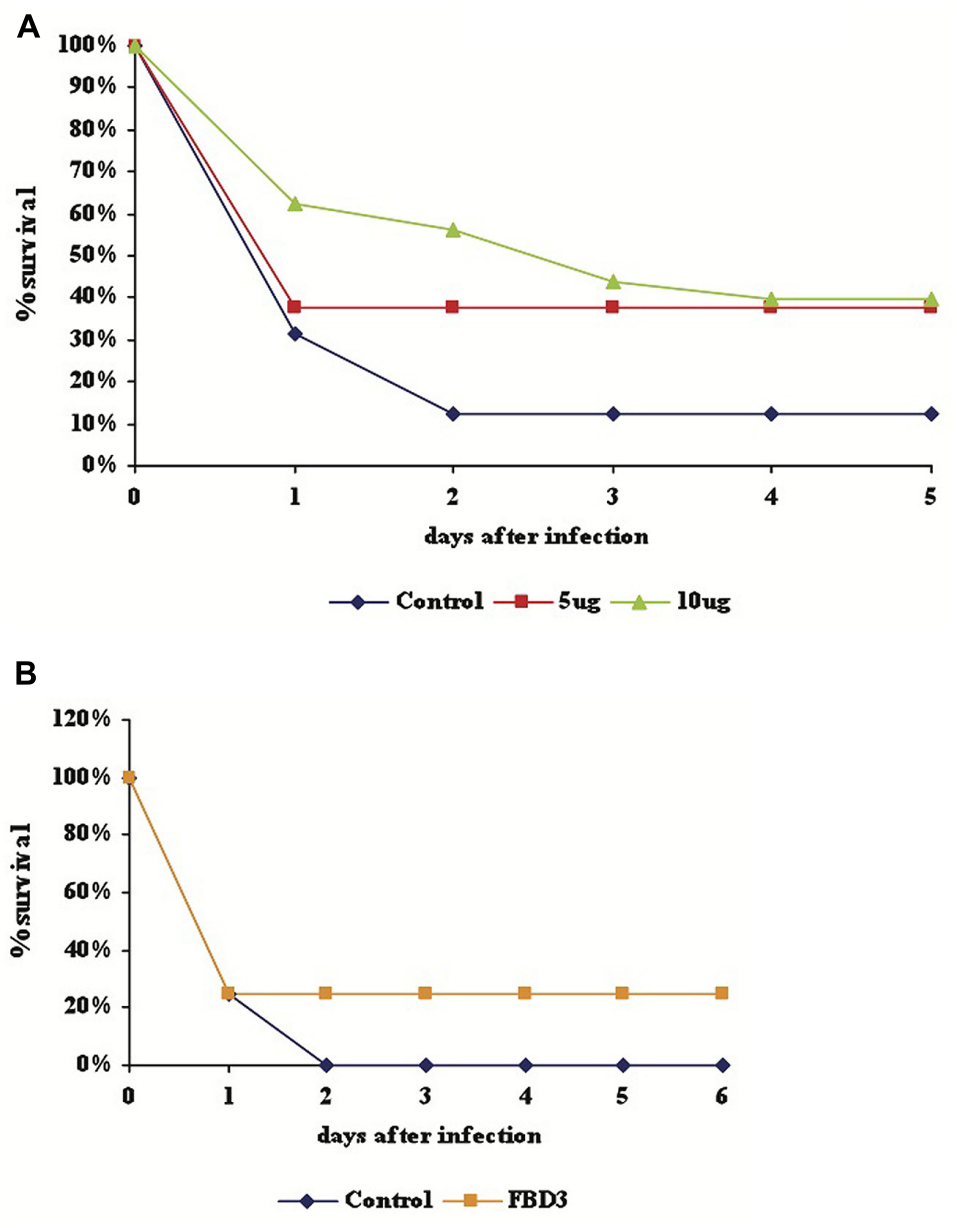
**Partially protective effect of F and FBD3 proteins on mice infected with pathogenic *E. coli* bacteria.** Seven to eight week old female Balb/C mice were injected i.p with purified F protein (5 μg; 8 mice or 10 μg; 10 mice) **(A)** or FBD3 fusion protein (20 μg; 10 mice) **(B)**. After 4 h 100 μl of 5 × 10^8^ CFU/ml of *E. coli* bacteria was injected i.p. into the mice. Mouse mortality was determined over the next 5 or 6 days. Control represents infected mice that received only PBS (in equal volume); each control group consisted on eight mice.

In order to test the ability of our proteins to protect mice from bacterial infection, purified F (5 or 10 μg), FBD3 (20 μg), or PBS (control, in equal volume) was injected into 7–8 week old female Balb/C mice, for a preliminary study. Four hours after i.p. injections of proteins/PBS, 100 μl of 5 × 10^8^ CFU/ml of pathogenic *E. coli* bacteria were injected i.p. into these mice. Mouse survival was followed over the next few days. As seen in **Figure 6A**, the F protein conferred a partial protective effect for the bacteria-infected mice. After 4 days the survival rate of the mice in the control group was 12.5% while the survival rate of the mice, which had received 5, or 10 μg of the F protein was 37.5 and 40% respectively.

As seen in **Figure 6B**, 20 μg FBD3 also had a partial protective effect on mice infected with pathogenic *E. coli* bacteria. No mice survived after day two in the control group, while the survival rate of the treatment group remained at 20% for the next five days. These very preliminary results indicate a potential partially protective effect of F and FBD3 proteins in bacteria-infected mice.

### THE MECHANISM OF FLAGELLIN’S ACTION ON CD4+ T CELLS

To reveal some basic aspects of flagellin’s action via its receptor, TLR5, we used mononuclear cells from healthy humans. We chose to test the effect of flagellin on the CD4+ T cell subpopulation, which has been suggested to be a bridge between the innate and the adaptive immune system through the TLR5 receptor expressed on these cells.

To test the events following flagellin stimulation, we used real time PCR to follow changes in mRNA levels of several genes known to play central roles in immune processes. CD4+ T cells were isolated from peripheral blood of healthy donors by affinity purification, using CD4 magnetic beads. We first checked the isolated CD4+ T cells for the expression of TLR5 using FACS analysis and found that 96% (mean: 129) of naïve CD4+ T cells express the TLR5 receptor (results not shown).

#### The immune system reaction – cytokine profile

**FIGURE 7:**
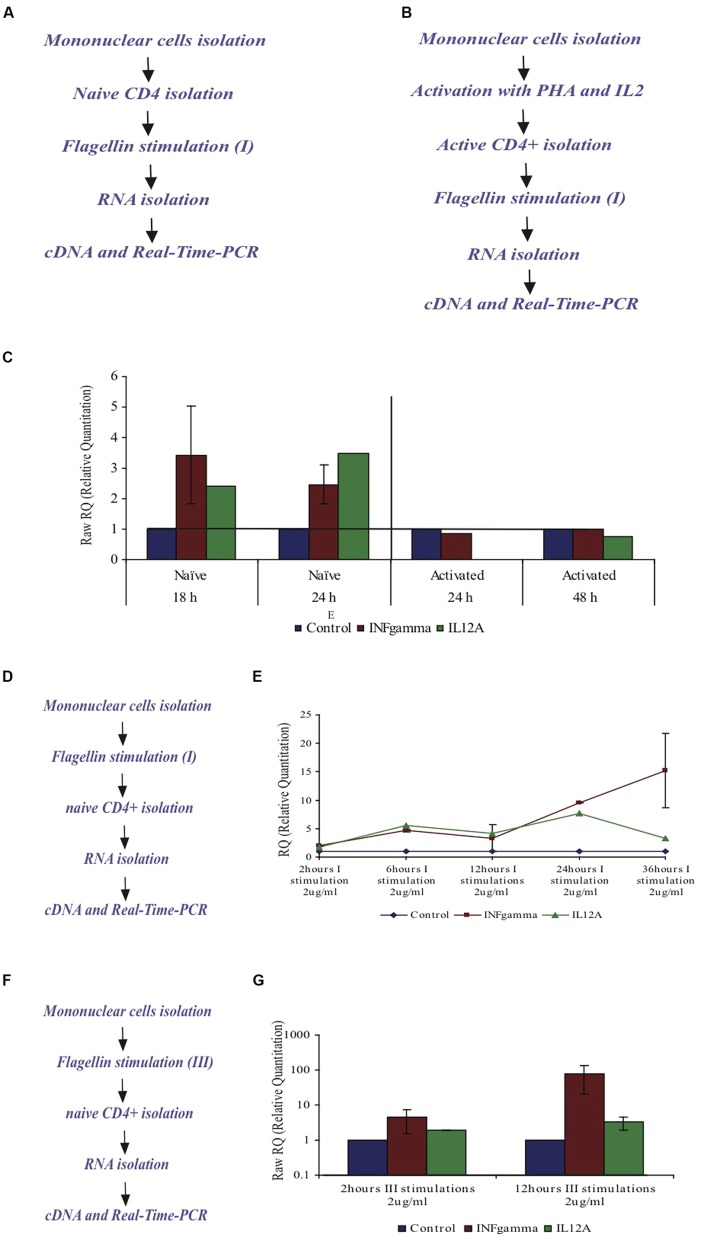
**Effect of F protein on expression levels of IL12A and IFNγ in CD4+ T cells from healthy donors. (A–C)** Effect of F protein on expression levels of IL12A and IFNγ in naïve and activated CD4+ T cells. **(A)** Experimental design for naïve cells – CD4+ T cells were isolated from peripheral blood of healthy donors using CD4+ magnetic-beads. CD4+ T cells were seeded and incubated with the F protein (2 μg/ml) for 18 or 24 h. **(B)** Experimental design for activated cells – mononuclear cells were activated by PHA (20 μg/ml) for 72 h and then grown with IL2 (10 units/ml recombinant IL-2). Following activation, CD4+ T cells were separated using CD4 magnetic beads. CD4+ T cells were seeded and incubated with the F protein (2 μg/ml) for 24 or 48 h. Following the incubation, cDNA samples were prepared and subjected to real-time-PCR analysis using SYBR green reagent (as described in Methods). **(C)** Changes in IFNγ-mRNA and IL12A-mRNA levels upon F stimuli, compared to control cells. I = one F stimulation. Data represents the mean ± SD of 3–5 separate experiments. **(D,E)** Effect of the F protein on expression levels of IL12A and IFNγ in naïve CD4+ T cells after triggering all mononuclear cells. **(D)** Experimental design – Naïve mononuclear cells were incubated with flagellin for different time periods, after which CD4+ T cells were separated using CD4 magnetic beads. The separated CD4+ T cells were taken and cDNA samples were prepared and subjected to real time-PCR analysis using SYBR green reagent (as described in Methods). **(E)** The changes of IFNγ-mRNA and IL12A-mRNA in CD4+ T cells levels upon flagellin stimuli in the presence of mononuclear cells after 2, 6, 12, 24, and 36 h. I = one F stimulation. Data represents the mean ± SD of 3–5 separate experiments. **(F,G)** Effect of the F protein on expression levels of IL12A and IFNγ in naïve CD4+ T cells after triggering the mononuclear cells three times. **(F)** Experimental design – Naïve mononuclear cells were triggered three times with flagellin for 2 or 12 h stimuli. Following stimulation, CD4+ T cells were separated using cd4 magnetic beads. The separated CD4+ T cells were removed and cDNA samples were prepared and subjected to real time PCR analysis using SYBR green reagent (as described in Methods). **(G)** Changes of IFNγ- and IL12A mRNA levels upon repeated flagellin stimulations for 2 or 12 h each. III = three F stimulations. Data represents the mean ± SD of 3–5 separate experiments.

***Flagellin affects naïve CD4+ T cells but not activated CD4+ T cells***. In order to test the cytokine profile upon flagellin stimulation, naïve CD4+ T cells were isolated from peripheral blood of healthy donors and triggered with F protein for various time periods (**Figure 7A**). We found that INFγ-mRNA levels increased by 3.4-fold after 18 h and by 2.4-fold after 24 h, as compared to control un-stimulated cells (**Figure 7C**). We also found that IL12A-mRNA increased by 2.5-fold after 18 h and by 3.5 after 24 h (**Figure 7C**). The mRNA levels of the genes for IL4 and IL5 in control, untreated samples were too low to allow calculation of the changes in the mRNA levels but based on the cycle’s number in the real time PCR analysis, we estimated that there was no change in the mRNA levels of these genes upon flagellin stimulation (results not shown). Thus, flagellin stimulation caused the CD4+ T cells to increase the mRNA levels of IL12A and IFNγ, the cytokines associated with the Th1 response.

Next we tested the effect of flagellin on activated CD4+ T cells. Whole mononuclear cells were isolated from peripheral blood of healthy donors, activated by PHA for 72 h and then grown in the presence of IL2 (10 units/ml recombinant IL-2) to maintain the activated phenotype. Activated CD4+ T cells were then isolated using CD4 magnetic beads and stimulated with flagellin for 24 or 48 h (**Figure 7B**). In the activated CD4+ T cells, the mRNA levels of the genes for IL12A and INFγ did not change upon flagellin stimulation (**Figure 7C**).

***The impact of the “neighbor cells” on cytokine profile after flagellin stimulation***. The expression of TLR5 has been described for many cell populations of the immune system in addition to T cells. These cells produce a variety of cytokines that may influence the CD4+ T cells. Because CD4+ T cells are not alone *in vivo*, we took whole mononuclear cells (after ficoll gradient) and stimulated them with F in an attempt to mimic the natural environment of T cells in the body. This allowed us to examine the contribution of “neighbor-cells” to the expression level of the different cytokines in CD4+ T cells, upon flagellin stimulation.

We triggered whole mononuclear cells with flagellin for 2, 6, 12, 24, and 36 h, then separated CD4+ T cells and isolated RNA to measure the expression levels of specific genes (**Figure 7D**). As seen in **Figure 7E**, stimulation with F protein for 2 h caused the mRNA levels of cytokines IL12A and INFγ to increase by 1.66 and 1.94-fold, respectively. After 6 h the increase was even more significant with the levels of IL12A-mRNA and INFγ-mRNA increasing by 5.5 and 4.6-fold, respectively, and staying unchanged for up to 12 h. A prolonged 24 h exposure to flagellin caused a more dramatic increase in the expression of both genes, with IL12A-mRNA increasing by 7.6-fold and INFγ-mRNA increasing by 9.6-fold. 36 h of flagellin stimulation caused a higher increase in INFγ-mRNA of 15-fold, whereas the IL12A-mRNA levels decreased and were only 3.2-fold higher than in control cells (**Figure 7E**).

As seen in **Figure 7E**, long exposure to F protein (36 h) in the presence of all mononuclear cells caused a dramatic increase in the IFNγ-mRNA levels in CD4+ T cells. To examine this phenomenon more closely, we triggered all mononuclear cells three times with F protein (**Figure 7F**) and measured the levels of IFNγ and IL12A mRNAs. We found that three, 2-h stimulations, caused an increase in the mRNA levels of IFNγ and IL12A by 4.4 and by 5.1-fold, respectively (**Figure 7G**). Three, 12-h stimulations with F protein caused a dramatic increase in the mRNA levels of IFNγ of 78-fold when compared to non-treated cells. The increase of the IL12A mRNA under these conditions was 3.3-fold (**Figure 7G**).

### FLAGELLIN AFFECTS TLR5 mRNA LEVELS IN NAÏVE CD4+ T CELLS BUT NOT IN ACTIVATED CD4+ T CELLS

**FIGURE 8:**
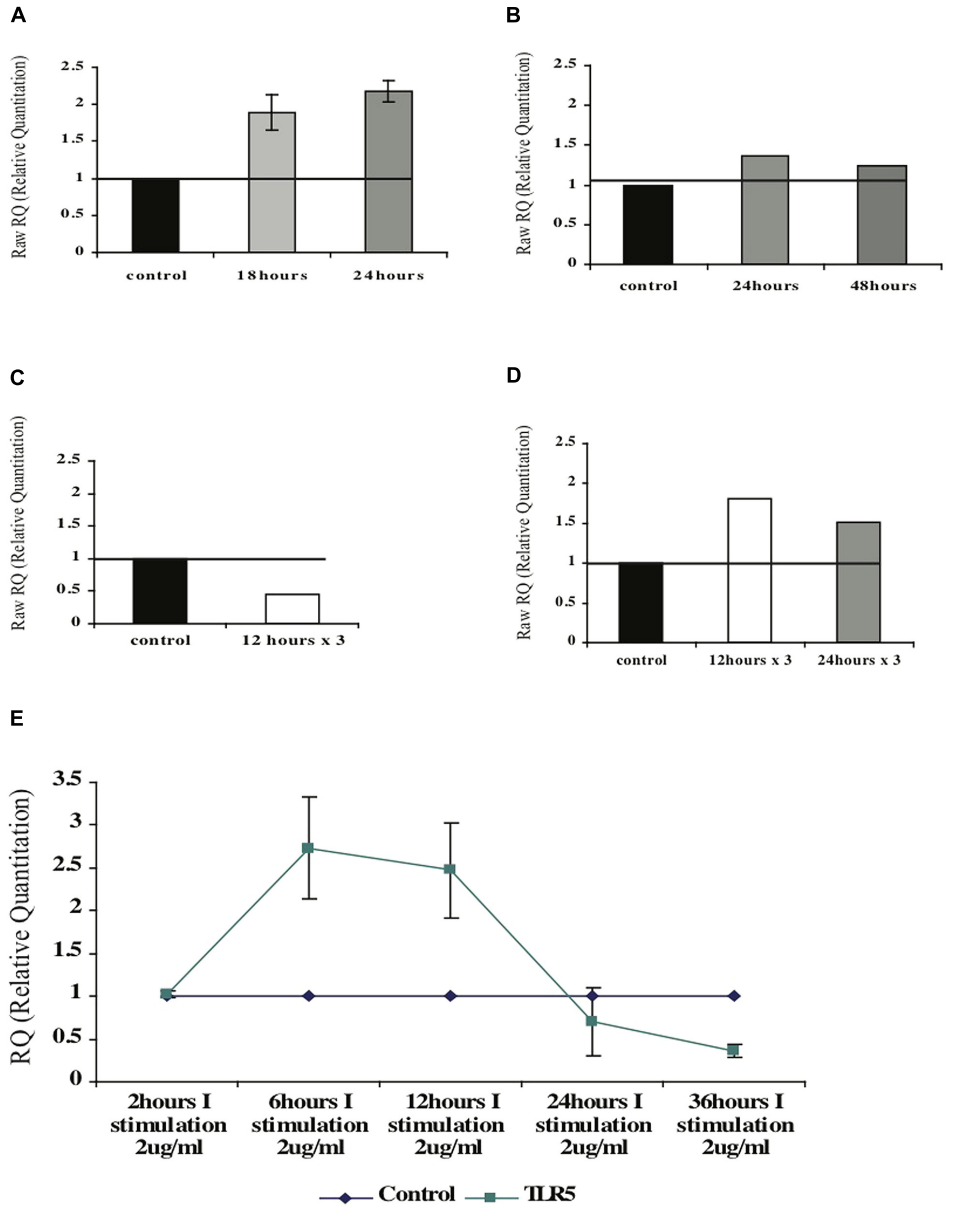
**Effect of the F protein on expression levels of TLR5 in healthy CD4+ T cells. (A,B)** Changes of TLR5-mRNA levels in naïve **(A)** or activated **(B)** CD4+ T cells upon one F stimulation after 18 or 24 h. **(C,D)** Changes of TLR5-mRNA levels following repeated (three) F stimulations in naïve **(C)** and activated **(D)** CD4+ T cells. **(E)** Changes of TLR5-mRNA levels following one F stimulation after various incubation times. Data for each graph represents the mean ± SD of 3–5 separate experiments **(A,B,E)** or one representative experiment out of two performed is shown **(C,D)**.

In order to examine whether flagellin has an influence on its own receptor TLR5, naive CD4+ T cells were isolated from peripheral blood of healthy donors and triggered with F protein for various time periods (see **Figure 7A**). We found that upon F stimulation the levels of the TLR5-mRNA increased by 1.8-fold after 18 h and 2.2-fold after 24 h as compared to control, unstimulated cells (**Figure 8A**).

Next, we tested whether flagellin triggering has the same effect on activated CD4+ T cells. CD4+ T cells were isolated from peripheral blood of healthy donors that was activated with PHA for 72 h (**Figure 7B**). Following activation the cells were stimulated with flagellin for different time periods. As seen in **Figure 8B**, stimulation with F protein for 24 or 48 h did not cause any significant increase in TLR5-mRNA levels in the activated CD4+ T cells. These findings are in correlation with our previous results regarding cytokines’ mRNA levels, showing that flagellin only affects the naïve CD4+ T cells (**Figure 7**).

#### The impact of continued flagellin stimulation on TLR5 mRNA levels in CD4+ T cells

We first tested isolated naïve CD4+ T cells. As opposed to a single trigger with F that caused an increase in the mRNA levels of TLR5, repeated stimulations for 12 h caused a twofold decrease in the TLR5-mRNA levels (**Figure 8C**). Activated CD4+ T cells, on the other hand, had slightly increased TLR5-mRNA levels upon repeated stimulations with F protein. Three stimulations of F, each for 12 or 24 h, caused an increase in the TLR5-mRNA levels by 1.8- and 1.5-fold respectively, as seen in **Figure 8D**.

These results indicate that naïve and activated CD4+ T cells react differently to flagellin triggering with respect to expression of the TLR5 receptor. We also observed that naïve cells are more sensitive to flagellin stimulation than activated cells; while naïve CD4+ T cells increased their mRNA levels after one stimulus, activated CD4+ T cells needed three stimulations to respond in a similar manner.

#### Neighboring cells have an impact on TLR5-mRNA levels in CD4+ T cells after flagellin stimulation

To test the impact of the neighboring cells on TLR5 regulation in CD4+ T cells, we used the total fraction of mononuclear cells and triggered them with F protein for various time periods (see **Figure 7D**). As seen in **Figure 8E**, 2 h of stimulation did not change the level of TLR5-mRNA but stimulation for 6 h increased the TLR5-mRNA by 2.8-fold a level at which it remained for at least 12 h. However, prolonged exposure to F protein caused the opposite result; 24 h of stimulation decreased the levels of TLR5-mRNA by 50%, with an even more significant decrease after 36 h of stimulation, of 3.5-fold lower than control untreated cells (**Figure 8E**).

### TLR5 AND INFγ – IS THERE ANY CONNECTION?

**FIGURE 9:**
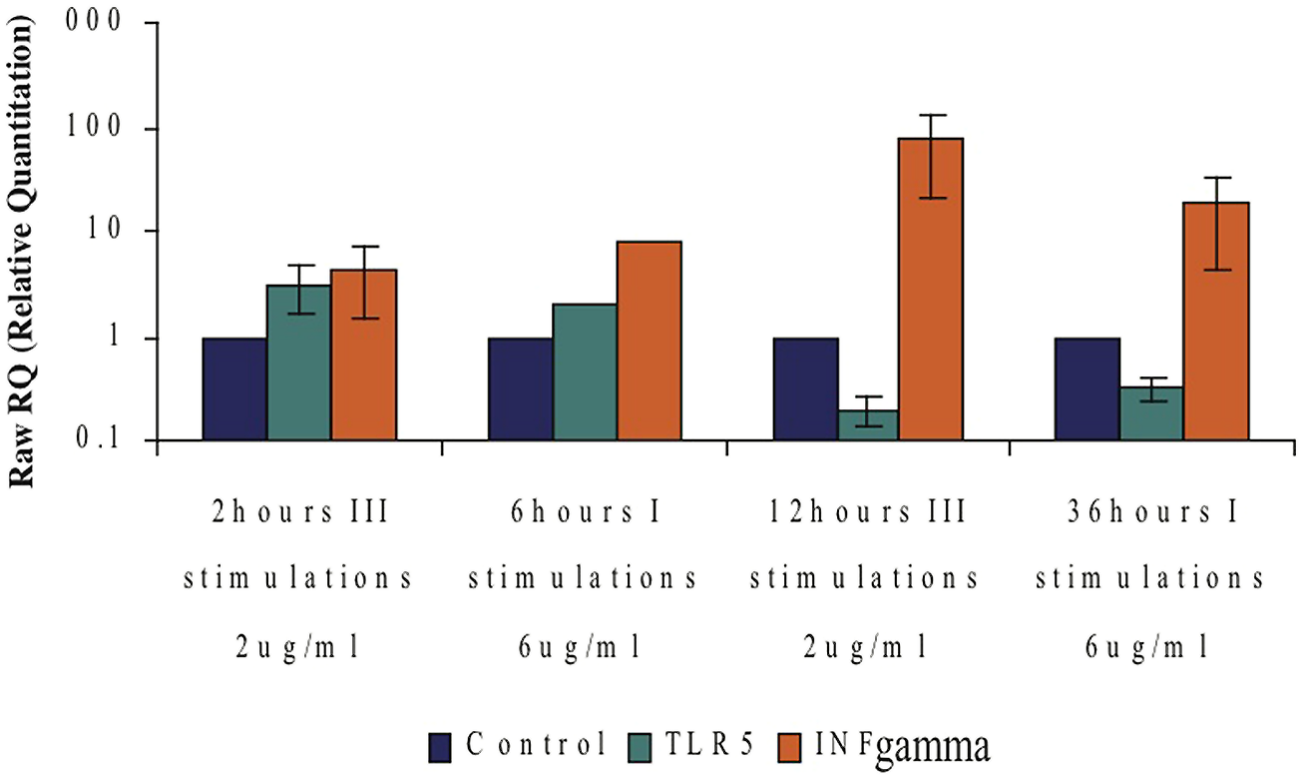
**Possible connection between the TLR5- and IFNγ-mRNA levels following flagellin stimulation.** Comparing expression levels of TLR5-mRNA to those of IFNγ-mRNA at different F protein stimulation conditions. Data represents the mean ± SD of three separate experiments.

An interesting connection between INFγ-mRNA levels and the TLR5-mRNA levels in CD4+ T cells was observed upon flagellin stimulation. Moderate levels of INFγ-mRNA were detected with increasing levels of TLR5-mRNA levels; however high levels of INFγ-mRNA were detected when the levels of TLR5-mRNA were very low (**Figure 9**). These results suggest a possible role of INFγ in the regulation of TLR5.

## DISCUSSION

One major concern in medicine today is the growing resistance to antibiotic treatment as a result of their widespread and intensive use (Spellberg et al., 2008) that has dramatically increased the emergence of resistant pathogens. The major threat is the spread of a virulent bacterium that is resistant to all antibacterial agents available today.

In this work we propose an innovative approach to fight bacterial infections based on the use of two components: the human antibacterial peptide BD3 that belongs to the innate immune system and the bacterial protein flagellin that induces the immune system. This combination was designed to provide an efficient weapon to attack bacterial infections; while the peptide kills the bacteria directly, the flagellin protein should trigger the immune system and act against residual bacteria that succeed in escaping the peptide’s action. In order to test this concept we used two strategies; the first was to design a fusion protein (FBD3) containing both molecules on one single polypeptide. The second strategy was to design the two components as separate molecules and use them either separately or in a combination.

We designed, expressed and purified the fusion protein FBD3, the recombinant protein F and the nBD3 peptide (**Figure 1**). We found that both the FBD3 fusion protein and the nBD3 peptide had antibacterial activity *in vitro* against several different bacterial strains [*E. coli* (DH5α), pathogenic *E. coli* (ATCC 25922), and *Staphylococcus aureus* (ATCC 6538)]; however with different efficacies (**Figures 2** and **4**). We also demonstrated that the flagellin moiety of FBD3 and the recombinant F protein, most probably, recognize their receptor, TLR5, which is expressed on target cells and confirmed their ability *in vitro* to specifically stimulate secretion of IL-8 (**Figure 5**). The F and FBD3 proteins were tested for their biological activity in an *in vivo* mice model; we showed that these proteins have a partial protective effect on mice infected by pathogenic *E. coli* bacteria that cause a lethal disease (**Figure 6**). In addition, we analyzed some basic mechanisms through which the F protein may be act, focusing on human CD4+ T cells from healthy donors.

The use of antibacterial peptides as therapeutic tools is limited since most of the peptides do not work at physiological concentrations of NaCl (Dhople et al., 2006). Additional limitations are the instability of the peptides and their toxicity for the host cells (Jenssen et al., 2006). However, the human BD3 is particularly attractive due to its strong antibacterial activity, relative salt-insensitiveness and low toxicity for host cells (Batoni et al., 2006). Indeed, nBD3 exhibits dose dependent antibacterial activity against a number of bacterial pathogenic strains (**Figures 2** and **3**). It should be pointed our that in our research, we cloned the nBD3 peptide without a tag and demonstrated that the purified peptide exhibited antibacterial activity (**Figure 2**). It should be emphasized that the expression of antibacterial peptides in bacterial expression systems is mostly achieved by tagging the peptide with various tags that are eventually cut out following expression and purification (Corrales-Garcia et al., 2011). The insertion of a tag to the peptide is done in order to protect the bacterial cells from toxic effects of the antibacterial peptide they are expressing. We decided to express the peptide without any tag due to the fact that most of the antibacterial peptide activity models suggest that the peptide works on the outer bacterial membrane (Brogden, 2005). The fact that we could express an active soluble peptide in a bacterial host was surprising and is in agreement with the proposed models of its activity (Brogden, 2005).

Flagellin and FBD3 proteins showed only a partial protective effect of about 20% in mice infected by pathogenic *E. coli* bacteria (**Figure 6**). This might be explained by the very severe and lethal infection induced in this model, as indicated by the very low (**Figure 6A**) or no survival (**Figure 6B**) of mice in the control, non-treated mice. We suggest that in a follow-up research, the efficacy of the F and FBD3 proteins should be tested *in vivo* on a much milder bacterial infection. In addition, experiments performed were preliminary, using only one bacterial strain (pathogenic *E. coli*) injected i.p. into mice as a route for inducing an infection. FBD3 and the F proteins should be tested in additional *in vivo* models to fully evaluate their therapeutic and protective potential.

However, It should be emphasized that we were able to show partial protection of mice infected with a pathogenic *E. coli* strain using a flagellin sequence from *Salmonella.* The fact that *Salmonella* flagellin has a protective effect against *Salmonella* infection in mice has been previously published (Vijay-Kumar et al., 2008). However, here we were able, to our knowledge for the first time, to protect mice from an *E. coli* infection using a flagellin sequence from *Salmonella typhimurium.* Our results thus suggest that the protective effect was most probably due to the activation of the innate immune system. The immediate and very fast protective response provides further findings supporting the idea that activation of the innate immune system was responsible for the protection (**Figures 6A,B**).

Moreover, our results suggest that flagellin or its derivatives such as FBD3 activate the innate immune response, and so may be used to treat bacterial infections. The innate immune response, in contrast to the adaptive response, is not specific to one specific antigen, so the ability to design antibacterial agents that will trigger only the innate immune system provides a breakthrough in medicine due to its broad spectrum of protection.

Nevertheless, to achieve a good and effective immune response, the innate and the adaptive immune systems work in collaboration. TLRs belong to the innate immune system, many cells however belonging to the adaptive immune system also express these receptors, suggesting that TLRs may serve as a bridge between the two components of the immune system. CD4+ T cells are a major subpopulation of cells that play a key role in the adaptive immune system and that express the TLR5. For this reason, we chose to focus on CD4+ T cells from healthy donors and to explore the effects of flagellin via its receptor TLR5 on various mechanisms.

As demonstrated by our results, only naïve CD4+ T cells respond to flagellin stimulation (**Figure 7**). The stimulation caused these cells to have increased mRNA levels of the cytokines IFNγ and IL12A that are characteristic of the Th1 response (**Figure 7**). However, a much more significant effect was achieved when we triggered the total mononuclear cell fraction with flagellin (**Figure 7**). Therefore, we suggest that flagellin has, most probably, both a direct and indirect effect on the cytokine profile in CD4+ cells.

Another interesting finding was the dramatic elevation in the IFNγ-mRNA levels caused by repeated flagellin stimulations (**Figure 7**). IFNγ is major cytokine that plays a central role in the immune response to infections (Saha et al., 2010). One of the observations in chronic diseases is the down regulation of the zeta chain of the T cell receptor (TCR). This phenomenon is related to sustained exposure to bacterial antigens (Bronstein-Sitton et al., 2003). TLRs play a major role in the induction of both the innate and the adaptive immune system. Previous studies have shown that several agonists belonging to the TLRs family lead to an immunosuppressive response; one of the mechanisms that were suggested as being responsible for this phenomenon is the secretion of IFNγ (Vaknin et al., 2008). These findings are relevant to our proposed approach, as they indicate that the number of flagellin stimulations will need to be carefully considered if flagellin therapy is further developed.

Constant stimulation of the immune system can be harmful for the host, with various inflammatory mediators causing damage (Bronstein-Sitton et al., 2003). To regulate the immune response many of the components of the immune system work through a feedback loop that helps the system to regulate itself and prevent autoimmune damage. Flagellin is a major antigen in many infections that can trigger the immune response by activating its receptor, TLR5 (Vijay-Kumar and Gewirtz, 2009). Therefore, we decided to test whether flagellin has a role in the regulation of this receptor. We demonstrated that isolated naïve CD4+ T cells triggered by flagellin increased their TLR5-mRNA levels, while activated CD4+ T cells did not respond (**Figure 8**). In addition, the naïve CD4+ T cells needed only one stimulus to respond, while the activated cells responded only after three stimuli (**Figure 8**). We also found that the neighboring cells have an effect on the regulation of TLR5-mRNA in CD4+ T cells; while a brief flagellin exposure caused an increase in TLR5 mRNA levels, a long exposure caused a decrease in the mRNA levels (**Figure 8**). From these findings we can propose a feedback role for flagellin in the regulation of its own receptor, TLR5. Short exposure to flagellin causes an increase in the mRNA levels of TLR5 and activates the immune response. To avoid continuing stimulation, long exposure to flagellin causes down regulation of the receptor. By this mechanism the immune system response can be regulated, without being self-damaging.

An additional interesting finding was the different response to flagellin seen in a isolated population of CD4+ T cells compared to that seen with a mixture of all the mononuclear cells. While isolated naïve CD4+ T cells exposed to flagellin for 24 h showed an increase in their mRNA levels, exposing total mononuclear cells to flagellin caused a decrease in the mRNA levels of TLR5 in the CD4+ T cells (**Figure 8**). We suggest that flagellin regulates TLR5, most probably, *via* both direct and indirect activity. In this regard, we found an interesting connection between the levels of the IFNγ and the levels of TLR5 upon flagellin stimuli; low levels of the TLR5 were detected when very high levels of IFNγ were measured (**Figure 9**). IFNγ is known to regulate the expression of a number of genes including MHCI genes, TLR3, STAT3, and many others (Saha et al., 2010). Thus, we suggest that IFNγ may have role in the regulation of the TLR5 gene.

We found that the flagellin moiety in the FBD3 fusion protein did not loss its activity and had high activity on colo-205 cells *in vitro* as the separate F protein (**Figure 5**). The observation that flagellin did not lose its function in the fusion protein is important for designing new additional fusion proteins based on flagellin.

For the antibacterial activity, our results indicated that nBD3 was active *in vitro* (**Figures 2** and **3**). The fusion protein was more difficult to produce. However, with regard to the use of the fusion protein, we also need to consider a molecule that contains a flagellin sequence lacking the hypervariable region. In addition to the lower toxicity of its flagellin moiety, this fusion protein will be smaller in size and this may prevent its negative impact on the peptide’s action. Thus we believe that both approaches, that of using the FBD3 fusion protein and that of utilizing the separate components, the F protein and the nBD3 peptide, should be further investigated and developed.

## Conflict of Interest Statement

The authors declare that the research was conducted in the absence of any commercial or financial relationships that could be construed as a potential conflict of interest.
